# Health insurance and health system (un) responsiveness: a qualitative study with elderly in rural Tanzania

**DOI:** 10.1186/s12913-021-07144-2

**Published:** 2021-10-22

**Authors:** Paul Joseph Amani, Anna-Karin Hurtig, Gasto Frumence, Angwara Denis Kiwara, Isabel Goicolea, Miguel San Sebastiån

**Affiliations:** 1 Department of Health Systems Management, School of Public Administration and Management, Mzumbe University, Morogoro, Tanzania; 2 Epidemiology and Global Health, Umeå University, Umeå, Sweden; 3 Department of Development Studies, School of Public Health and Social Sciences, Muhimbili University of Health and Allied Sciences, Dar es Salaam, Tanzania

**Keywords:** health insurance, elderly, responsiveness, healthcare use, Tanzania

## Abstract

**Background:**

Health insurance (HI) has increasingly been accepted as a mechanism to facilitate access to healthcare in low and middle-income countries. However, health insurance members, especially those in Sub-Saharan Africa, have reported a low responsiveness in health systems. This study aimed to explore the experiences and perceptions of healthcare services from the perspective of insured and uninsured elderly in rural Tanzania.

**Method:**

An explanatory qualitative study was conducted in the rural districts of Igunga and Nzega, located in western-central Tanzania. Eight focus group discussions were carried out with 78 insured and uninsured elderly men and women who were purposely selected because they were 60 years of age or older and had utilised healthcare services in the past 12 months prior to the study. The interview questions were inspired by the domains of health systems’ responsiveness. Qualitative content analysis was used to analyse the data.

**Results:**

Elderly participants appreciated that HI had facilitated the access to healthcare and protected them from certain costs. But they also complained that HI had failed to provide equitable access due to limited service benefits and restricted use of services within schemes. Although elderly perspectives varied widely across the domains of responsiveness, insured individuals generally expressed dissatisfaction with their healthcare.

**Conclusions:**

The national health insurance policy should be revisited in order to improve its implementation and expand the scope of service coverage. Strategic decisions are required to improve the healthcare infrastructure, increase the number of healthcare workers, ensure the availability of medicines and testing facilities at healthcare centers, and reduce long administrative procedures related to HI. A continuous training plan for healthcare workers focused on patients´ communication skills and care rights is highly recommended.

**Supplementary Information:**

The online version contains supplementary material available at 10.1186/s12913-021-07144-2.

## Introduction

The world has witnessed a rapid increase in population ageing, particularly in low and middle-income countries (LMICs). Currently, about 5 % of the rural population in Sub-Saharan Africa (SSA) consists of people 60 years old and older [1]. In Tanzania, which has a total population of 45 million, there are roughly 2.7 million people (6 % of the total population) in this age group; this percentage is projected to increase to 11 % in the next three decades [2]. Over 80 % of this population resides in rural areas where there is restricted access to social services, including healthcare [3]. This demographic transition is creating a challenge regarding healthcare that requires urgent attention.

To face this situation, many countries in SSA have, including Tanzania, implemented different healthcare reform strategies with the aim of improving the availability, accessibility, and delivery of quality public health services to all people, including the elderly [4]. Beginning in the mid-1990 s, Tanzania witnessed the introduction of health insurance (HI) as a means to redress the ever-increasing expenditures on healthcare costs, and thus to protect the people from catastrophic healthcare expenditures [5]. Two HI models, the Community Health Fund (CHF) and the National Health Insurance Fund (NHIF), were implemented in 1996 and 2001 respectively. Currently, about 24 % of the Tanzanian population is covered under CHF and 6 % under the NHIF. CHF remains a voluntary insurance targeting the informal and rural population nationwide [6]. Through the CHF Act of 2001 [7], the government of Tanzania declared CHF a voluntary prepayment scheme and directed district councils administration to define the benefit package and a flat rate premium per year [8]. As of today, enrolment in CHF membership covers up to six household members [7]. Each household obtains a single membership card valid for 12 months that can be used in primary and secondary healthcare facilities within the district of registration. NHIF was originally compulsory for the government sector employees but was expanded in 2013 to cover the informal sector as a voluntary scheme [9]. Similar to CHF, NHIF covers up to six people in a family, but it provides coverage to a wider range of benefits in all healthcare system levels and across the country. Those elderly who retired from the public sector and had already joined NHIF would continue to be covered until death. While the average enrolment of CHF varies across districts, the 30 % coverage rate that was expected by the year 2015 has not yet been achieved. This might have been caused by diverse factors such as the voluntary nature of the schemes, a limited benefit package, and restricted use at local primary and secondary health facilities [10]. In addition to these insurance schemes, elderly people, considered to be poor to join the CHF, can apply to district social welfare officer for an exemption of costs related to health care at the public facilities within the district of residence [9].

Experience from SSA shows that HI has achieved mixed results. Studies from Ghana [11] and Rwanda [12] have shown that HI made access to care more equitable across the general population and reduced the burden of healthcare costs among the poor. Other studies from Ghana and Senegal [13, 14] have shown that the insured population aged 60 years and older were able to access inpatient and outpatient care services more than the uninsured. However, there are also negative experiences with HI; a study from Ghana showed that HI failed to protect its members equally, benefiting the educated, rich and those residing closer to health facilities more than others [15]. Findings from a qualitative study from the same country that explored the factors contributing to low uptake and renewal of HI cards revealed that a lack of leadership, poor communication, and the inability to pay the premium discouraged enrolment among community members [16]. Another study exploring the impact of social health insurance on the quality of care among women in Gabon revealed complaints about health staff rudeness, shortage of drugs, and non-coverage of essential medicines [17].

In Tanzania, several quantitative and mixed method studies on HI [5, 18] have shown an increased use and acceptability of HI by informal sector workers, particularly those residing in rural areas. However, challenges such as low enrolment, delayed reimbursement, unavailability of medicines and long waiting times [19], poor staff attitudes and limited or no diagnostic equipment [20], high premium for the poor, and limited referral services [21] have been reported. These findings have also been supported by other qualitative studies focused on understanding the views of the general population regarding the performance of HI [6, 22]. However, there is a lack of studies specifically looking at the role of HI among the elderly in the country, especially from a qualitative perspective.

Our own research has shown that even if HI can improve the use of healthcare among rural residents aged 60 years and older, it can also decrease the perception of responsiveness of the care [23]. In order to understand this lower responsiveness, this study aimed to explore the experiences and perceptions of healthcare services from the perspective of insured and uninsured elderly in rural Tanzania.

## Methodology

### Study setting

This was an explanatory qualitative study conducted in the Igunga and Nzega districts of the Tabora region, located in western-central Tanzania. Both districts are rural and have an estimated population of 901,979, of which 50,547 (6 % in Nzega and 5 % in Igunga) are elderly people aged 60 years and older [24]. These districts were chosen because of logistical reasons: the districts are next to one another and have large elderly populations. The situation of the health care system in these districts is comparable to any rural district in Tanzania with inadequate infrastructure and resources. In both districts, there exist primary and secondary healthcare levels where the elderly can access both outpatient and inpatient services regardless of their insurance status. The elderly who joined NHIF as civil servants continue to enjoy a lifelong coverage in both public and private facilities. CHF is a voluntary health insurance for rural households and their dependants and services are limited to public facilities and private accredited facilities within the district of registration. The insured elderly are entitled to free services such as consultation, prescriptions, surgical services, physiotherapy and rehabilitation services, and optical and dental health services [23].

### Study respondents

We conducted eight Focus Group Discussions (FGD), four in each district. The participants were purposely selected with the help of the hamlet leaders and two local research assistants. Inclusion criteria to participate in the study were: being 60 years of age or older, residing in one of the two districts, and had utilised healthcare services in the past 12 months prior to the study. A total of 90 potential participants were approached and 78 individuals (41 men and 37 women) with a mean age of 70 years participated. Of all the participants, 41 had health insurance, 38 of which were insured with the CHF.

### Data collection

The FGDs were conducted at one of the hamlet leaders’ offices or homes. Each FGD consisted of 9 to 12 participants and lasted between 45 and 65 min. Data collection ended when the research team felt that there was enough information to answer the research questions. Two FGDs included only insured elderly (FGD 1 & 2), two non-insured participants (FGDs 7 & 8), and four were mixed (FGDs 3–6). The FGDs were guided by a set of open-ended questions that aimed to prompt discussion regarding how participants perceived the quality of the healthcare services and to what extent the ownership of health insurance influenced the way the healthcare services responded to their needs. The structure of the interview guide was inspired by the seven domains of the WHO responsiveness framework [25]: quality of basic amenities, dignity, confidentiality, choice, prompt attention, communication, and autonomy (Appendix 1). Probes were used to motivate the discussion, to foster understanding, and to explore new topics. The FGD guide was revised after being piloted with three elderly who did not form part of the study participants. All the FGDs were conducted in Swahili, the mother language of the participants, the first author, and the moderator. The discussions were digitally recorded and transcribed verbatim and later translated into English language in order to facilitate analysis within the research group. Field notes were taken during the discussion and used during the analysis. The three domains of the COREQ (COnsolidated criteria for REporting Qualitative research) checklist were followed in the study (Appendix 2).

### Data analysis

Qualitative content analysis as proposed by Graneheim and Lundman [26] was used to analyse the transcripts of the FGDs. The steps in content analysis include summing the information into condensed meaning units, coding them, building categories from the codes, and making analytical interpretive connections between the categories [27]. Through the open coding process, key issues from the transcripts were identified as they related to the study question. All of the authors read each transcript to become familiar with the data. At first, we coded the data and inductively sorted the quotations into preliminary groups that described the experience of the elderly people with the healthcare system and how they perceived the health insurance to affect these experiences. Then, we connected the developed codes with the domains of responsiveness [25]. We grouped the codes into the following related domains: quality of basic amenities, dignity, confidentiality, choice, prompt attention, communication, and autonomy. A new category – “access” – was constructed because it did not correspond with any of the responsiveness domains and covered aspects related to access to medicines and laboratory facilities. The freely available open-code software was used to support the coding process [28]. The first author (PA) developed the first codes from the data and then worked with IG to create subcategories and categories of codes. Through several meetings, all authors discussed the coding process, interpretation, and trustworthiness of the findings.

### Ethical considerations

 The research and ethical committee (REC) at the Muhimbili University of Health and Allied Sciences (MUHAS) approved the research protocol. Permission for data collection was obtained from the District Executive Directors of the Igunga and Nzega districts. Written consent to participate in the study was obtained from all the participants. Before conducting each FGD, the aim and the scope of the study were explained prior to soliciting the consent of participants. Participants were also informed about their right to withdraw from the study anytime and their permission to audiotape the discussions. Based on the nature of the discussions within focus groups, we explained to the participants that it would be challenging to guarantee complete confidentiality. Thus, participants were requested not to comment upon the issues discussed outside the FGD and were assured that the data would be presented in a manner that would preserve anonymity.

## Results

Seven categories capturing the different responsiveness domains were developed, as conceptualised by WHO: buildings appearance is valued (*quality of basic amenities*), disrespectful care is still common (*dignity*), keeping patients’ information safe is a challenge (*confidentiality*), insurance reinforces inequality of care (*choice*), increased bureaucracy and few human resources lead to increased waiting time (*prompt attention*), patient-doctor relationship is important (*communication*), and being involved in the care decision process is difficult (*autonomy*). An additional category, accessibility of medicines and testing facilities should be improved (*acces*s), originated from the data. These categories describe the experiences of the elderly people with the healthcare system and the role of the HI in meeting (or not meeting) the expectations regarding their interactions with the system.

### Buildings appearance is valued

The participants discussed the state of the facilities’ infrastructure where services were obtained in great detail. First of all, they recognised that there had been some efforts by the government to improve the hospital buildings and health facilities in the district in order to enhance access and delivery of healthcare services. However, participants were mostly concerned with the inadequate space and general cleanliness, not only inside the healthcare facilities but also in the surroundings, as the following quotations describe:

*“…There are some old buildings with small few offices, wards, and toilets facilities which need renovation because they make the hospital look not clean…”* (FGD 2).

*“…In some facilities, the environment is not adequately cleaned, sometimes the cleaning is done while patients are already at the hospital and also some patients drying their clothes on the walls and on the grass…”* (FGD 3).

One positive achievement that was highlighted was the allocation of a specific place within the hospital area for attending elderly people. However, the insured participants discussed the wish of having a specific place accommodated to them to facilitate a better service. As one participant mentioned:

*“…I wish that there could be a building a bit large for the insured elderly people only. This is because the designated place is too small and there are so many elderly people…”* (FGD 4).

### Disrespectful care is still common

The participants considered it important to comprehensively discuss their experience on the type of attention healthcare workers provided when they interacted with the healthcare system. Several participants appreciated the way they were treated at the healthcare facilities and reflected on improvements over time:

*“…We currently thank God for having some health workers who give their services with all commitment; they care and respect us. They don’t look down on us as they used to in the past…”* (FGD 1).

Even many insured participants recognised that the health staff’s practices towards them had improved:

*“…Some health workers care and respect us insured elderly…when we get to the hospital, doctors and nurses run towards us”* (FGD 1).

However, the participants considered a number of issues disrespectful. Many of them complained that HI made no difference in the way they were considered and treated as patients. Some insured participants expressed their disappointment when they realised that the HI did not guarantee that they would receive different and better attention:

*“…You could get there very sick and providers wouldn’t care; you have no value. Medical personnel just pass around without doing anything…they despise the health insurance service, even when they listen to you they don’t pay much attention…”* (FGD 7).

A few of them mentioned that being insured could even negatively influence the type of care provided. This is described by one informant who reflected on how the healthcare professional addressed him when he came in seeking healthcare:

*“…Old man you are here again with your CHF, you never become tired, we don’t accept these cards here…”* (FGD 2).

### Keeping patients’ information safe is a challenge

Ensuring confidentiality in the process of care was widely discussed in the FGDs; participants explained that it constituted a challenge for all patients, insured or not. However, several participants reacted positively to the development of this issue over time:

*“…Confidentiality is not a big problem today; it has been improved [and is] now different from the past…currently we are talking inside the room with the door closed…”* (FGD 3).

*“…Today confidentiality has been ensured [and] information about the patient doesn’t leak…Whatever you talk to the doctor remains inside the room…it is well kept by some officers…”* (FGD 1).

However, some elderly also reflected about certain attitudes from health workers that they considered to be a violation of their privacy:

*“…Some of the doctors would stop and talk to the patient along the corridor and start to ask you [client] about your problems, illnesses or sometimes they read your [clients’] test results in front of other people and patients. Sometimes you could enter a doctor’s room and he/she starts to interrogate while the door was open and other people would be listening…”* (FGD 3).

The participants also expressed concerns about the potential risk of confidentiality breaks when attending private providers:

*“…If you go to buy medicines out of the hospital then it is likely that your information may leak because who gives you medicines obviously knows about your sickness…”* (FGD 1).

### Insurance reinforces inequalities of care

The topic of referrals was mainly discussed among insured participants. While members of the CHF had expected a greater degree of coverage when another health facility outside their registration district was attended, members of the NHIF acknowledged that the insurance enabled them to choose service providers and get referrals to higher levels of care across the country:

*“…A person with insurance [NHIF]) has free access to healthcare facility; even when medicine is not available, he/she can get it at a private store. Honestly…we had to take her [my wife] to Muhimbili National hospital [Dar es Salaam] because my insurance [NHIF] could pay for the services…”* (FGD 3).

Differences in access clearly appeared between the types of insurance when individuals were referred to other districts with higher healthcare capacity. As the following quote shows, CHF was not helpful when referrals to other districts were needed:

*“…My wife was treated here at Igunga, then was transferred to Ocean Road Hospital [Dar es Salaam]. Although we had the CHF insurance, it just helped here at Igunga, but when we reached Ocean Road Hospital things changed and with great costs…”* (FGD 5).

Some CHF members even reflected on the possibility of withdrawing from the scheme due to the lack of coverage in the outside-district health services:

*“…I want to do away with CHF because it is only useful (…) in the district. Outside this district, it cannot be accepted…[or] used…. I have requested my children to help me join the National Health Insurance scheme so that I can be able to access the services across the country and in good hospitals”* (FGD 7).

### Increased bureaucracy and few human resources lead to increased waiting time

The elderly were asked about their perception regarding prompt attention for healthcare services. Several participants commented that waiting time had improved recently despite the small number of health providers, particularly for those with health insurance:

*“…The quality of health services has been improved…doctors push themselves to help despite being few in number…but waiting time is not bad especially if you have a health insurance”* (FGD 1).

But others indicated that the shortage of health professionals contributed to longer waiting times and less time allocated with the doctor, thus affecting the quality of care.

*“…It is true maybe that the number of human resources at many of our facilities are few in numbers. By simple observation, you realise that they take a very long time to attend [to] few patients… If there were more doctors, things would go very fast…”* (FGD 2).

Some elderly complained that having health insurance did not necessarily mean a shorter waiting time because of the bureaucratic process they have to follow regarding registration and access to medicines and laboratory services:

*“…When you enter there with your insurance you will take more time waiting for services starting from the registration….and as well on medicines you may be told to wait while they search their availability…”* (FGD 7).

*“…It is hectic sometimes with the CHF. Registration takes [a] long [time because staff have] to get the patients’ files. You may arrive at the health facility at nine in the morning and get out three or four in the afternoon if you are lucky…and with your CHF you cannot get services, medicines, or even laboratory tests quicker…”* (FGD 1).

### Patient-doctor relationship is important

The role of effective communication in the process of care was viewed as important by both insured and non-insured participants, but their experiences regarding the provision of a clear explanation to their health problem were mixed. Some insured elderly noticed some improvement:

*“…When prescribing you some medicines he will elaborate to you what are they for and their side effects…”* (FGD 3).

*“…Currently…when you are with the doctors explaining your problems, you talk, he/she listens and responds back, and even when you make mistakes you are corrected…”* (FGD 5).

However, other participants, including those with HI, were concerned about the lack of a proper communication between the patient and the provider:

*…In fact we [CHF] members came in large numbers and the doctors are few…the doctors could not have much time to spend to listen to each patient and understand…before you explain the doctor had already written the prescription for you…”* (FGD 6).

*“…You reach there as a patient and at the same as an insured elderly person, the providers just toss you around like football. Go there for registration….until the day ends because you cannot pay cash…”* (FGD 4).

### Involvement in the care decision process is far from happening

The need to provide information to individuals about their health status, risks, and treatment alternatives is a core domain of the responsiveness framework. Two main challenges were revealed from the FGDs regarding the involvement of the patient in the care process. The first one is related to how participants experienced the lack of health patient’s involvement in their care-decision process, as the following quotes exemplify:

*“…If you are lucky, you might be asked about the kind of treatment or medicines you normally take….but in general, honestly it is the doctor who decides which medicine you should take and decides on every other thing…”* (FGD 2).

*“…There is a chance for you to share your views, but let us tell the truth, there is no involvement in decision making here…”* (FGD 5).

The second challenge, which is related to the first, was that the amount of patients and the shortage of personnel were reported as factors that contribute to the lack of involvement at the consultation:

*“…I don’t understand how we can be involved in the process while doctors are not even [plentiful] enough to attend us…”* (FGD 5).

### Accessibility of medicines and testing facilities should be improved

An additional category not included in the WHO responsiveness domains resulted from the discussion: the challenges of accessing medicines and diagnostic facilities. Some participants thought that improvements had been achieved over the years:

*“…In the past there used to be very poor services whereby we could not get the prescribed medicines…but after the government insisted that medicines should be available right at the hospitals, these services have been improved…although I have not been to the hospital often, but I went there and honestly they gave me all the necessary services…”* (FGD 1).

*“…To tell the truth, for the days when I went there at the hospital the laboratory services were not bad…”* (FGD 4).

However, most agreed that the shortage of these important resources at the health center and hospital levels was common. As a consequence, they were instructed by the health workers to obtain such resources in private drugstore and laboratories, which often implied the possibility of not having access to them at all:

*“…We are always told that there is no medicine or sometimes we get half a dose and the rest we are told to go and buy from private pharmacies…”* (FGD 1).

*“…In most cases, some of the elderly went back home without buying the medicine because they do not have the financial capacity to buy [it]”* (FGD 4).

To be insured under the NIHF was generally considered an advantage regarding access to medicines. If they were not available, healthcare staff provided them with a permit and referred them to obtain the medicines at an accredited pharmacy:

*“…We [NHIF] get medicines and when they don’t have them they give us forms which we use to get medicines at Chacha’s pharmacy”* (FGD 6).

*“…The insured normally is given prescription[s] for medicine at the hospital; if [it is] not available, a form is filled in so that they can go and get it at the pharmacy [NHIF]….[for] those under CHF, once they miss the medicine they are supposed to buy on their own from the pharmacies…”* (FGD 4).

Participants also complained that, on occasion, the CHF was neither helpful nor an advantage to the elderly regarding medicines and testing services, as they often ended in private costly facilities that they had to pay themselves:

*“…Some medicines are expensive and I think that is why they are not available in the hospitals…I tell you we elderly are going to suffer while holding our insurances….because the prescribed medicines is more expensive than we use to pay for insurance…*(FGD 1).

*“…When it is a bit advanced like full blood picture, dialysis etc., they ask you to go [to] private laboratories in town. There you use your money and it is very expensive because they do not accept CHF…”* (FGD 3).

A specific complaint was raised by several insured participants concerning the attitude of some healthcare staff who favored patients who paid in cash instead of those with insurance when collecting medicine:

*“…I came here knowing that I would be attended because I have my insurance card but providers at the medicine window refuse it and they say we don’t accept these CHF cards…”* (FGD 2).

## Discussion

This explanatory qualitative study provided insight on both health insured and non-insured elderly in rural Tanzania regarding both their experiences with and perceptions of healthcare, as well as their expectations on how the health system should take care of them. Specifically, the study revealed the role of HI in facilitating the fulfilment of those expectations. In general, HI was considered to be a useful tool that enabled access to healthcare. However, the perspectives of the elderly varied widely across the explored domains of responsiveness. In addition, assuring accessibility to medicines and testing facilities was impeded due to the constant shortage of these resources. It is important to mention here that the exemption for healthcare cost policy is a useful strategy to enhance access to healthcare by the rural poor elderly. Nevertheless, while this policy was not discussed in the interviews, we can hypothesize that all challenges experienced by those with HI could as well apply to those under the exemption policy. The discussion is structured according to three topics that summarise the main challenges of the HI from the perspective of the elderly: (1) HI as a safety net; (2) HI as a foster of inequity; and (3) HI as a promoter of low responsiveness.

### Health insurance as a safety net

One of the key functions of the HI is to protect the people against the increasing healthcare costs and thus facilitate their access to services [7]. Several participants in the study felt HI provided this protection and thereby reduced barriers to the use of healthcare services.

These findings are in line with HI studies from within and outside Tanzania. From inside the country, a study looking at seeking behaviour and utilisation of healthcare by members of different insurance schemes noticed an increase in healthcare seeking due to reduced financial obstacles [29]. A recent study by Kapologwe et al. [30] found that HI attracted timely visits to healthcare facilities, while another study exploring the views of one rural community regarding CHF [18] showed that HI was well accepted as an alternative mechanism to reduce out-of-pocket payments Along the same lines, another study reported that CHF members had a greater possibility of being financially protected against catastrophic expenditures than non-insured members [31]. Similarly, a study assessing the role of HI coverage in maternal health services in Tanzania showed that insured women missed less of their first antenatal visits and delivered more of their children in the hospital compared to non-insured women [32]. Several studies from SSA have also shown that HI has the potential to provide risk protection, thereby reducing the burden of healthcare costs among the poor [33] and making it a key mechanism for achieving universal health coverage [34].

However, some of the participants in the study perceived that HI led to unexpected healthcare costs, mostly due to the shortage of medicines and laboratory services. Similar findings were seen in a study that explored the distribution of healthcare financial burden in Tanzania, which concluded that the poor received the lowest share of the benefits precisely because CHF failed to cover expensive hospital services [5]. Another study found that primary healthcare facilities in Tanzania commonly encounter a shortage of drugs and other essential supplies, thus forcing the insured to pay at private pharmacies [6]. The additional payments despite having a CHF may discourage members of the scheme from seeking healthcare on time [18]. Studies from Ghana and South Africa have shown that out-of-pocket payments in healthcare are still prevalent among insured households [35, 36].

### Health insurance as a foster of inequity

Enabling equal access to healthcare independent of one’s socioeconomic status is a top priority of HI in the country. Studies on the role of HI on healthcare equity are becoming important in understanding how different insurance schemes influence healthcare utilisation, choice of service providers, and financial protection [37, 38]. Although little is known about equity in healthcare in Tanzania, findings from a recent study by our research group suggested that both horizontal and vertical equity in access of both outpatient and inpatient care by the insured rural elderly [39].

The difference in service coverage that exists between the two current public health insurances, CHF and NIHF, however, creates a unfair discrepancy regarding service utilisation among its members. This situation was revealed by the participants’ complaints regarding the unequal access to healthcare depending on the insurance scheme. This has also been noticed in a study that found that the benefits obtained by CHF members were restricted to the primary and secondary healthcare facilities within the districts of registration [9]. In addition, another study that explored the risk distribution across CHF and NHIF schemes in Tanzania warned that the presence of multiple funds with different benefits packages and serving diverse population groups could promote an inequitable use of healthcare services [40].

Similar findings have been reported across LMICs regardless of the insurance model in operation. For instance, experience from Jordan showed that members of the Ministry of Health insurance had a higher probability of seeking care compared to other insurance schemes [41]. Similarly, healthcare inequalities have been reported in China among subgroups of insurance enrollees [37]. In an Armenian study assessing the equity of community health insurance, membership was found to be low as a result of the lack of affordability and the offer of a primary healthcare package that did not cover chronic diseases nor referrals to higher levels of care [42]. Similarly, in a study in Krygyzstan and Moldova, the relatively well-off members were found to benefit more from the HI, leaving the poor to access limited and poor quality services [43].

### Health insurance as promoter of low responsiveness

Although HI seemed to facilitate service utilisation, different challenges regarding certain dimensions of the health system’s responsiveness were highlighted. These have been organised into two main areas: one related to the providers’ attitudes in the patient-health worker relationship, and one related to the organisation of the healthcare system. Several dimensions of the WHO framework on responsiveness such as dignity, autonomy, confidentiality, and communication directly relate to the attitudes of the healthcare providers towards their patients. While the majority of participants did make comments related to these dimensions, comments from those that had HI were more noticeable. This has also been observed, for instance, in a recent study from the same area where a better performance of the responsiveness domains was reported among non-insured elderly as compared to insured [23]. Our findings are also similar to other studies from Tanzania, albeit not related to the elderly, that reported an increasing patient dissatisfaction with the negative attitude of healthcare staff, which insured patients referred to as discourteous, impolite, and unhelpful [6, 44].

Certain similarities and differences with studies from LMICs that have focused on the elderly and health insurance are also worth discussing. For example, in a study from Nigeria [45], the interaction of insured patients and providers resulted in a reported low responsiveness for dignity but relatively high responsiveness for communication and involvement in decision-making. In Brazil [46], elderly patients were satisfied with dignity, confidentiality, and communication, but not with autonomy; in South Africa, the elderly reported dissatisfaction with their involvement in decision-making, communication, and respect domains [47].

Knowledge of healthcare providers regarding patients’ right to be treated with respect is an important aspect of the Tanzanian healthcare system that is emphasised through various guidelines and policies including the National Health policy of 2017 [48] and the National Ageing policy of 2003 [49]. However, reported observations of disrespectful care continue to challenge the primary healthcare in the country [50]. Similarly, providers in Ethiopia and South Africa have been blamed for not treating the elderly with respect [51].

Our findings also reveal the failure of the Tanzanian health system, and HI in particular, to enable patients to be involved in their treatment decisions, highlighting an important information asymmetry within the healthcare system. This has also been found in studies from Nigeria and South Africa that reported the elderly’s low involvement in their individual healthcare decisions [45, 47].

While a lack of confidentiality was revealed in the discussions, studies from both within and outside the country tend to point in another direction, where the elderly are usually satisfied with the way their private information is safeguarded [30, 52].

 The participants’ need and demand for better communication with their providers was an important finding. Patients’ demands for more time and a clearer explanation of their diagnosis and treatment are commonly found in the literature [45, 53].

Numerous efforts have been made in the country to improve the basic practice of health professionals through formal training, seminars, and workshops, and by offering standard operating procedures through supportive supervision at council levels since 1999 [54]. However, this work does not seem to be adequate, at least in rural areas.

The quality of basic amenities and prompt attention relates to the way the healthcare system is organised and influences the process of service delivery. As previously discussed, a lack of these dimensions creates a barrier for accessing the services, particularly among the insured elderly. A proper infrastructure is a prerequisite for patients to feel welcome. While the government of Tanzania has initiated the Primary Health Services Development Program (2007–2017), among other programmes, to rehabilitate and upgrade the infrastructure of primary health facilities [55], several studies in the country have reported that these facilities are deteriorating [56, 57]. Different studies have insisted that the adequacy of hospital infrastructure is a prerequisite for the successful implementation of HI in LMICs [58, 59]. A lack of hygiene and basic amenities cannot only put patients at risk, but also cause them to refrain from joining HI or attend healthcare services more generally [51].

Patient waiting time is a growing concern for healthcare managers and policy-makers in Tanzania. The elderly participants in this study expected that HI could accelerate this process but it did not; in some extreme cases, participants were not given any attention whatsoever. One study from Tanzania identified long waiting times and delays in administrative processes as a barrier of accessing care, regardless of HI status [60]. Similarly, long waiting times have been reported as a common problem across SSA countries, particularly in the case of HI [61]. From our findings, it seems that the benefits of HI are heavily hindered by the generally bleak conditions of the country’s health system.

### Methodological considerations

Several strengths and limitations of the study must be considered. The informants were purposely chosen based on their ability to contribute to the study’s aim. In order to enhance the quality of trustworthiness, several measures were taken. An emergent design using an interview guide based on our earlier studies from the same setting and topic was developed. To increase credibility, categories were developed and quotations from the scripts were provided to illustrate that our interpretations remained grounded on the data. Both men and women participated in the FGD and measures to ensure that men did not dominate the discussions and that women’s voices were acknowledged and listened to were taken by the moderator. Since the FGDs were conducted in Swahili and later translated into the English language to allow the participation of the other authors, it is possible that some important meanings from the original narration got lost in the translation process. However, the involvement of the first author in the FGDs who is familiar with the language and culture of the respondent increased the credibility of the study’s findings. We have also described the characteristics of the setting so that the reader can judge how transferable our findings are to other similar rural settings.

## Conclusions

To our knowledge, this is the first explanatory qualitative study that explores the experiences and perceptions of elderly people (both insured and uninsured) regarding the responsiveness of the healthcare system in rural Tanzania. Elderly participants appreciated that HI had facilitated their access to healthcare and protected them from certain healthcare costs, but they also complained that HI had failed to provide equitable access due to limited service benefits and restricted use of services within schemes. Elderly perspectives varied widely across the domains of responsiveness.

While promoting HI can be a good strategy for the elderly to access healthcare, several efforts should be taken by the government to make this a reality. CHF policy should be revisited in order to improve its implementation, expand the scope of services coverage, and, where possible, to introduce cross-subsidisation between the publicly owned schemes. More strategic policy decisions are required to improve the healthcare infrastructure, increase the number of healthcare workers, ensure the availability of medicines and testing facilities at healthcare facilities, and reduce long administrative procedures related to HI. A continuous training plan for healthcare workers focused on patients’ communication skills and care rights is highly recommended. Further qualitative research with other stakeholders, including health professionals, to understand their perspective on HI will be important to carry out in the near future.

## Supplementary information

**Additional file 1**

**Additional file 2**

## Data Availability

The dataset that was generated and analysed in this study is not publicly available owing to protect the confidentiality of the respondents, but is available from the corresponding author on reasonable request.

## Abbreviations

CHF: Community Health Fund
FGD: Focus Group Discussion
HI: Health Insurance
LMIC: Low and Middle Income Countries
MUHAS: Muhimbili University of Health and Allied Science
NHIF: National Health Insurance Fund
REC: Research and Ethical Committee
SSA: Sub-Saharan Africa
WHO: World Health Organization
