# Supplementary material for: Health insurance and health system (un) responsiveness: a qualitative study with elderly in rural Tanzania
Source: BMC Health Serv Res. 2021 Oct 22;21:1140. doi: 10.1186/s12913-021-07144-2 (PMC8532322; doi:10.1186/s12913-021-07144-2)
Supplement: Supplementary file 1 — Additional file 1 [file 12913_2021_7144_MOESM1_ESM.docx]

Appendix 1. FGD Guide questions for elderly

a) How has your health been the last year?

b) Have you had the need to go to any health care facility?

c) How was your experience when you went there?

Probe: availability of services, referral process and system, privacy, waiting time, availability of human resources (e.g. geriatricians ), payment mechanisms, communication with providers, involvement in decision making, accreditation process etc.

d) How did you deal with the costs derived from care? Did your insurance scheme cover all the costs?

e) What do think about health insurance? (Positive and negative aspects of being insured)
