# Supplementary material for: Health insurance and health system (un) responsiveness: a qualitative study with elderly in rural Tanzania
Source: BMC Health Serv Res. 2021 Oct 22;21:1140. doi: 10.1186/s12913-021-07144-2 (PMC8532322; doi:10.1186/s12913-021-07144-2)
Supplement: Supplementary file 2 — Additional file 2 [file 12913_2021_7144_MOESM2_ESM.docx]

**Appendix 2. COREQ (Consolidated criteria for Reporting Qualitative research) Checklist**

| **Topic** | **Item No.** | **Guide Questions/Description** | **Reported on Page No.** |
| --- | --- | --- | --- |
| **Domain 1: Research team and reflexivity** |  |  |  |
| *Personal characteristics* |  |  |  |
| Interviewer/facilitator | 1 | Which author/s conducted the interview or focus group? | PA (page 8, line 174-175) |
| Credentials | 2 | What were the researcher’s credentials? E.g. PhD, MD | Master |
| Occupation | 3 | What was their occupation at the time of the study? | Researcher/Lecturer |
| Gender | 4 | Was the researcher male or female? | Male |
| Experience and training | 5 | What experience or training did the researcher have? | The first author has received extensive training on qualitative methodologies as part of his PhD process. All team members have considerable experience in conducting qualitative research (please refer to page 27 line 596). |
| *Relationship with participants* | | |  |
| Relationship established | 6 | Was a relationship established prior to study commencement? | Not. However, with the help of the hamlet leader and research assistants it was possible to come into contact and then recruit the elderly for the FGDs (please refer to page 7 line 149) |
| Participant knowledge of the interviewer | 7 | What did the participants know about the researcher? e.g. personal goals, reasons for doing the research | They had known the goal and reasons for the research |
| Interviewer characteristics | 8 | What characteristics were reported about the inter viewer/facilitator? e.g. Bias, assumptions, reasons and interests in the research topic | The reasons and interest in the research topic as a PhD student and that I was familiar with the language and culture of the respondents (please refer to page 27 line 606-607) |
| **Domain 2: Study design** |  |  |  |
| *Theoretical framework* |  |  |  |
| Methodological orientation and Theory | 9 | What methodological orientation was stated to underpin the study? e.g.  grounded theory, discourse analysis, ethnography, phenomenology, content analysis | Qualitative content analysis proposed by Graneheim and Lundman (26) (please refer to page 1 (line 36) and page 9 (line 181) |
| *Participant selection* |  |  |  |
| Sampling | 10 | How were participants selected? e.g. purposive, convenience, consecutive, snowball | The participants were purposely chosen based on their potential to the research topic [please refer to pages 1( line 33), 7 (line 155) and 27 (596)] |
| Method of approach | 11 | How were participants approached? e.g. face-to-face, telephone, mail, email | They were contacted  face to face |
| Sample size | 12 | How many participants were in the study? | In total there were 78 (41 men and 37 women) who participated in 8 FGDs |
| Non-participation | 13 | How many people refused to participate or dropped out? Reasons? | In total, we recruited around 90 elderly who did not participate in the survey. But only 78 showed up for the discussion. 12 participants did not show up (4 invitees had travelled outside the village, 3 said they were sick and 4 requested not to participate). No single participant dropped out during the discussion (please refer to page 8, line 158) |
| *Setting* |  |  |  |
| Setting of data collection | 14 | Where was the data collected? e.g. home, clinic, workplace | The FGDs were conducted at one of the hamlet leaders’ offices or homes (please refer to page 8 line 164) |
| Presence of nonparticipants | 15 | Was anyone else present besides the participants and researchers? | Yes, there was two research assistants (refer to page 7 line 156) |
| Description of sample | 16 | What are the important characteristics of the sample? e.g. demographic data, date | Mean age 70yrs, used health services in the past 12 months |
| *Data collection* |  |  |  |
| Interview guide | 17 | Were questions, prompts, guides provided by the authors? Was it pilot tested? | Yes, was piloted and revised accordingly (please refer page 8 line 174). |
| Repeat interviews | 18 | Were repeat inter views carried out? If yes, how many? | None |
| Audio/visual recording | 19 | Did the research use audio or visual recording to collect the data? | Yes. All the FGDs were audio recorded (please refer to page 8 line 177) |
| Field notes | 20 | Were field notes made during and/or after the interview or focus group? | Yes. Notes were taken during and after the discussion and were referred to during the analysis (please refer to page 8 line 179) |
| Duration | 21 | What was the duration of the inter views or focus group? | The FGDs took between 45 and 65 minutes (please refer to page 8 line 165) |
| Data saturation | 22 | Was data saturation discussed? | Yes, within the research team |
| Transcripts returned | 23 | Were transcripts returned to participants for comment and/or |  |
| **Topic** | **Item No.** | **Guide Questions/Description** | **Reported on Page No.** |
|  |  | correction? | No, we did not do that |
| **Domain 3: analysis and findings** |  |  |  |
| *Data analysis* |  |  |  |
| Number of data coders | 24 | How many data coders coded the data? | 1 |
| Description of the coding tree | 25 | Did authors provide a description of the coding tree? |  |
| Derivation of themes | 26 | Were themes identified in advance or derived from the data? | Yes, from the data (please refer to page 9, line 185-199) |
| Software | 27 | What software, if applicable, was used to manage the data? |  |
| Participant checking | 28 | Did participants provide feedback on the findings? | Not yet, but we have arranged a dissemination meeting on the 24-25th August 2021 with elderly people, regional and district health management teams and insurance officials in the districts where data was collected. |
| *Reporting* |  |  |  |
| Quotations presented | 29 | Were participant quotations presented to illustrate the themes/findings?  Was each quotation identified? e.g. participant number | Yes it was done (please refer to page 9 line 189-191) |
| Data and findings consistent | 30 | Was there consistency between the data presented and the findings? | Yes. We maintained this throughout the analysis process (please refer to page 9 line 198-200) |
| Clarity of major themes | 31 | Were major themes clearly presented in the findings? | Yes, in our case, categories (please refer page 9 line 185 -200) |
| Clarity of minor themes | 32 | Is there a description of diverse cases or discussion of minor themes? | While developing the categories, we developed subcategories to guide the description of each category however, these sub categories are not presented in the manuscript. |

Developed from: Tong A, Sainsbury P, Craig J. Consolidated criteria for reporting qualitative research (COREQ): a 32-item checklist for interviews and focus groups. *International Journal for Quality in Health Care*. 2007. Volume 19, Number 6: pp. 349 – 357
